# Wounds of the Air Passages

**Published:** 1898-05

**Authors:** 


### WOUNDS OF THE AIR PASSAGES.

*Charlotte Medical Journal* thus summarizes the treatment of wounds of the air passages: (1) Suicidal wounds of the throat should be treated by primary suture in all cases where the general condition of the patient permits. (2) Antiseptic precautions are most important. (3) If necessary, chloroform should be administered, and is perfectly safe. (4) Divided muscles should be sutured, and in bringing together the edges of the skin the inversion caused by the platysma muscle should be corrected. (5) The wound in the air passage should be completely closed. (6) In many cases it is quite safe to dispense with the use of a tracheotomy tube. If the tube be deemed necessary, it should not be introduced through the suicidal wound in the air passage, but through a fresh vertical cut at a lower level. (7) Silk is the best material for suturing the larynx or trachea. (8) During the after treatment it is necessary, except in certain special cases, to feed by a tube or by the rectum. (9) If the above methods of treatment be adopted, not only will a very large proportion of even dangerous and extensive wounds of the air passages recover, but the period of recovery will be greatly shortened, the patient will not be exposed to the same risks of secondary inflammatory complications. He will be much less liable to the occurrence of 'permanent stenosis' of the trachea, or the formation of an aerial fistula.—*Kansas Medical Journal.*

